# Real-world Cohort Study on the Effectiveness and Safety of Filgotinib Use in Ulcerative Colitis

**DOI:** 10.1093/ecco-jcc/jjad187

**Published:** 2023-12-08

**Authors:** Beatriz Gros, Mairi Goodall, Nik Plevris, Nathan Constantine-Cooke, Alexander T Elford, Claire O’Hare, Colin Noble, Gareth-Rhys Jones, Ian D Arnott, Charlie W Lees

**Affiliations:** Edinburgh IBD Unit, Western General Hospital, Edinburgh, UK; Department of Gastroenterology and Hepatology, Reina Sofía University Hospital, Córdoba, Spain; Maimonides Institute of Biomedical Research [IMIBIC], Córdoba, Spain; Medical School, University of Glasgow, Glasgow, UK; Edinburgh IBD Unit, Western General Hospital, Edinburgh, UK; MRC Human Genetics Unit, Institute of Genetics and Cancer, University of Edinburgh, Western General Hospital, Edinburgh, UK; Centre for Genomics and Experimental Medicine, Institute of Genetics and Cancer, University of Edinburgh, Western General Hospital, Edinburgh, UK; Edinburgh IBD Unit, Western General Hospital, Edinburgh, UK; Faculty of Medicine, University of Melbourne, Melbourne, VIC, Australia; Edinburgh IBD Unit, Western General Hospital, Edinburgh, UK; Edinburgh Pharmacy Unit, Western General Hospital, Edinburgh, UK; Edinburgh IBD Unit, Western General Hospital, Edinburgh, UK; Edinburgh IBD Unit, Western General Hospital, Edinburgh, UK; Centre for Inflammation Research, Queen’s Medical Research Institute, University of Edinburgh, Edinburgh, UK; Edinburgh IBD Unit, Western General Hospital, Edinburgh, UK; Edinburgh IBD Unit, Western General Hospital, Edinburgh, UK; Centre for Genomics and Experimental Medicine, Institute of Genetics and Cancer, University of Edinburgh, Western General Hospital, Edinburgh, UK

**Keywords:** Ulcerative colitis, small molecule, filgotinib, inflammatory bowel disease

## Abstract

**Background:**

Filgotinib is a small molecule with preferential inhibition of Janus kinase type 1, approved for the treatment of ulcerative colitis in Scotland in May 2022. We present the first real-world experience on its use in clinical practice.

**Methods:**

In this retrospective, observational, cohort study we assessed patients with active ulcerative colitis who received filgotinib in NHS Lothian, Scotland. Baseline demographic, phenotype, and follow-up data were collected via review of electronic medical records.

**Results:**

We included 91 patients with median treatment duration of 39 weeks (interquartile range [IQR] 23-49). Among the cohort, 67% [61/91] were biologic- and small molecule-naïve, and 20.9% [19/91] had failed one and 12.1% [11/91]  two or more classes of advanced therapy. Of the biologic- and small molecule-naïve patients, 18% [11/61] were also thiopurine-naïve. Clinical remission [partial Mayo score <2] was achieved in 71.9% [41/57] and 76.4% [42/55] of patients at Weeks 12 and 24 respectively. Biochemical remission [C reactive protein ≤5 mg/L] was achieved in 87.3% [62/71] at Week 12 and 88.9% [40/45] at Week 24. Faecal biomarker [calprotectin <250 µg/g] remission was achieved in 82.8% [48/58] at Week 12 and 79.5% [35/44] at Week 24. At the end of follow-up, median 42 weeks [IQR 27-50], 82.4% [75/91] of patients remained on filgotinib. Severe adverse events leading to drug discontinuation occurred in 2.2% [2/91] and there were 8.8% [8/91] moderate adverse events that required temporary discontinuation.

**Conclusion:**

These are the first reported data on the real-world efficacy and safety of filgotinib in ulcerative colitis. Our findings demonstrate that filgotinib is an effective and low-risk treatment option for these patients.

## 1. Introduction

Filgotinib, a small molecule with preferential inhibition of Janus kinase [JAK] type 1, received approval for the treatment of ulcerative colitis [UC] in Scotland in May 2022.^1^ Its approval was based on the positive outcomes from the phase 2b/3 SELECTION trial programme, demonstrating both efficacy and safety in UC^2^ with rapid and sustained symptom relief demonstrated in post hoc analysis.^3^

JAKs, a group of four intracellular tyrosine kinases, namely JAK1, JAK2, JAK3, and TYK2, play a pivotal role in governing various cellular processes, including inflammatory pathways. They are responsible for promoting the functions of lymphocytes and cytokines and are critically involved in essential bodily functions like haematopoiesis and defence against viral infections.^4^

The rationale for developing filgotinib lies in its specific affinity to JAK1, distinguishing it from the previous available small molecule tofacitinib, which exhibits a non-specific affinity to JAK1, JAK3, and at higher doses to JAK 2 and TYK 2, earning its label as a pan-JAK inhibitor.^5^ The hypothesis is that a more selective inhibition of JAKs will induce fewer adverse events while maintaining its efficacy. Although there are indirect comparisons between these molecules, a direct head-to-head trial has not been done.^6^

Despite the promising results from clinical trials, to date, no real-world data have been published yet concerning filgotinib’s efficacy and safety in UC. However, filgotinib has been used in other immune-mediated diseases for which there are available data, such as rheumatoid arthritis.^7,8^

In the Edinburgh IBD Unit, filgotinib started to be used from July 2022, with a significant number of UC patients receiving it as a first-line treatment. The aim of this study was to assess the real-world effectiveness and safety of filgotinib in UC, while also identifying predictive factors associated with favourable treatment response. This study aims to provide valuable insights that complement existing clinical trial data, assisting clinicians in making informed decisions regarding the use of filgotinib in UC.

## 2. Methods

### 2.1. Study design

We performed a retrospective, observational, cohort study performed at three sites within NHS Lothian [Western General Hospital, Royal Infirmary Hospital, and St Johns Hospital]. NHS Lothian provides universal, free at point of care, health care for a population of 916, 310 people [2022], including a rigorously validated prevalent population of 10, 499 patients with inflammatory bowel disease [IBD] in 2019.^9^

### 2.2. Participants

We identified all adult [>18 years old] patients with UC receiving filgotinib from July 2022 to May 2023, via pharmacy and electronic medical health records [TrakCare patient management ©InterSystems] with follow-up until 22nd September 2023. Inclusion criteria were a confirmed diagnosis of UC or IBD unclassified [IBD-U] favouring UC [based on standard criteria], and active disease. Patients with Crohn’s disease, IBD-U favouring Crohn’s disease, microscopic colitis, or with extensive colonic resection, were excluded.

### 2.3. Data collection

Baseline demographic, phenotyping, and follow-up data were collected via review of electronic medical records. Data regarding: clinical disease activity scores [partial Mayo score]; C-reactive protein [CRP]; faecal calprotectin [FC]; dose adjustments; steroid prescriptions; hospitalisation rates; and adverse events were collected. All FC samples were measured using a standard enzyme-linked immunosorbent assay [ELISA, Calpro AS™, Norway].

### 2.4. Definitions

Clinical remission, biochemical remission, and faecal biomarker remission were defined as a partial Mayo <2, CRP ≤5 mg/L, and FC <250µg/g, respectively. Normal albumin level was defined as ≥36 g/L. Primary non-response was defined as ongoing disease activity as evidenced by an elevated partial Mayo score [≥2] and/or elevated biochemical and faecal biomarkers [CRP >5 mg/L/FC ≥250µg/g] within 12 weeks, resulting in treatment discontinuation. Secondary loss of response with was defined as initial clinical and/or biomarker remission with subsequent development of an elevated partial Mayo score [≥2] and/or elevated biochemical and faecal biomarkers [CRP >5 mg/L/FC ≥250µg/g], resulting in treatment discontinuation. Comorbidity data were collected pertaining to heart conditions, previous thrombotic event, immune-mediated diseases, diabetes mellitus, and liver disease. Steroid prescription during follow-up was defined as prednisone or budesonide prescribed at any point after filgotinib induction. Serious adverse events were defined as any event leading to permanent discontinuation of therapy, hospitalisation, or death; moderate adverse events were defined as any event requiring temporary discontinuation, with all other adverse events defined as mild.

### 2.5. Primary and secondary outcomes

The primary outcome of this study was clinical remission at Week 12 [±4 weeks]. Secondary outcomes included: biochemical and faecal biomarker remission at Week 12 [±4 weeks], clinical remission, biochemical remission, and faecal biomarker remission at Week 24 [±8 weeks]; change in partial Mayo, CRP, and FC during follow-up; drug persistence; hospitalisation rates; baseline predictors of filgotinib persistence; baseline predictors of clinical remission at Week 12 and adverse events.

### 2.6. Statistical analysis

SPSS Version 25 [IBM, Armonk, NY] was used for statistical analyses and generation of graphs. Descriptive statistics are presented as medians with interquartile range [IQR] for continuous variables, and frequencies with percentages for categorical variables. For comparison of non-parametric continuous variables, the Mann–Whitney U or Kruskall–Wallis test was used where appropriate, and the Wilcoxon test for paired data. For comparison of categorical variables, the chi square test was used. Survival analysis was performed using Kaplan–Meier analysis, and comparisons made using the log-rank test. Patients were censored at last follow-up. For the effectiveness outcomes, analysis was performed on patients with available data. Cox proportional hazard regression analyses were carried out to identify possible baseline predictors of drug survival. Variables for analysis were chosen a priori [Supplementary Table 1]. Variables from the univariable analysis with a *p*-value <0.10 were fitted, and entered the multivariable analysis. A *p*-value <0.05 was considered significant. Variables associated with clinical remission at Week 12 were examined using logistic regression analysis.

### 2.7. Ethics

This work was considered a clinical service evaluation, as all data were collected as part of routine clinical care. Therefore, no written consent or formal ethical approval was necessary as per departmental policy and Health Research Authority.^10^

## 3. Results

### 3.1. Study population

A total of 91 patients met the inclusion criteria, 51.6% [47/91] were female, with a median age at treatment initiation of 42 years [IQR 32-56]. The median disease duration was 7 years [IQR 3-14 years]. Disease extent as per Montreal classification was: E1 14.4% [13/91], E2 44.5% [40/91], and E3 41.1% [37/91].

Of the 91 patients, 67% [61/91] were biologic- and small molecule-naïve, and 20.9% [19/91] had failed one and 12.1% [11/91] two or more classes of advanced therapy. Of the biologic- and small molecule-naïve patients, 18% [11/61] were also thiopurine-naïve.

Pre-treatment endoscopy [within the previous 8 weeks] was performed in 56% [51/91] patients, of whom 86.3% [44/51] demonstrated mild-moderately active disease [Mayo endoscopic score 1-2] and 13.7% [7/51] moderate to severe [Mayo endoscopic score 3]. The majority of patients had moderately active disease clinically at baseline observation, with a median partial Mayo score 5 [3-7]. In addition, median CRP was 3 g/L [1-11] and median FC was 780 µg/g [425-1166] [Table 1]. Regarding treatment dosing, 97.8% [89/91] received 200 mg/day and two patients received 100 mg/day due to chronic kidney disease. At the time of filgotinib initiation, 39.6% [36/91] were on steroids and 76.9% [70/91] were on 5-aminosalicylates [5-ASA] [Table 2].

**Table 1.** Pairwise comparison between baseline and Week 12 clinical, biochemical and fecal biomarker parameters.

*N *= 91	Week 0	Week 12	*p*
CRP, mg/L, median [IQR]	3 [1-11]	1[1-3]	<0.0001
Albumin, g/L, median [IQR]	37 [35-39]	39 [37-41]	<0.0001
FC, ug/gr, median [IQR]	780 [425-1166]	103 [25-195]	<0.0001
Total cholesterol, mmol/L, median [IQR]	4.9 [4.1-5.5]	5.1 [4.5-5.9]	0.25
Alanine transaminase, IU/L, median [IQR]	18 [12-25]	17 [13-25]	0.15
Partial Mayo score, median [IQR]	5 [3-7]	1 [0-2]	<0.0001

CRP, C-reactive protein; FC, faecal calprotectin; IQR, interquartile range.

**Table 2:** Baseline characteristics of patients.

*N *= 91	
Age, years, median [IQR]	42 [32-56]
Female, *n* [%]	47 [51.6]
Disease duration, years, median [IQR]	7 [3-14]
Disease extent, *n* [%]	
- Proctitis	13 [14.4]
- Left-sided	40 [44.5]
- Extensive	37 [41.1]
Extra intestinal manifestations, *n* [%]	23 [25.8]
Previous therapies	
Thiopurine, *n* [%]	36 [39.6]
Anti TNF, *n* [%]	18 [19.8]
Vedolizumab, *n* [%]	15 [16.5]
Ustekinumab, *n* [%]	4 [4.4]
Tofacitinib, *n* [%]	7 [7.7]
Naïve to biologic/small molecule, n [%]	61 [67]
Naïve to biologic/small molecule and thiopurine, *n* [%]	11 [12.1]
Filgotinib dose 200mg/d, *n* [%]	89 [97.8]
Concomitant steroids, *n* [%]	36 [39.6]
- Prednisone, *n* [%]	21 [23.1]
- Budesonide, *n* [%]	10 [11]
- IV methilpred, n [%]	5 [5.5]
Concomitant 5-ASA, *n* [%]	70 [76.9]
Baseline partial Mayo ≥2, *n* [%] * Missing data = 3	80 [90.9]
Baseline CRP >5 mg/L, *n* [%] * Missing data = 2	35 [38.5]
Baseline FC ≥250 µg/g, *n* [%] * Missing data = 16	65 [86.7]
Baseline albumin <36 g/L, *n* [%] * Missing data = 7	29 [34.5]

AntiTNF, anti-tumour necrosis factor; CRP, C-reactive protein; FC, faecal calprotectin; IQR, interquartile range; 5-ASA, 5-aminosalicylates; d, day..

### 3.2. Efficacy outcomes

**Figure 1:**
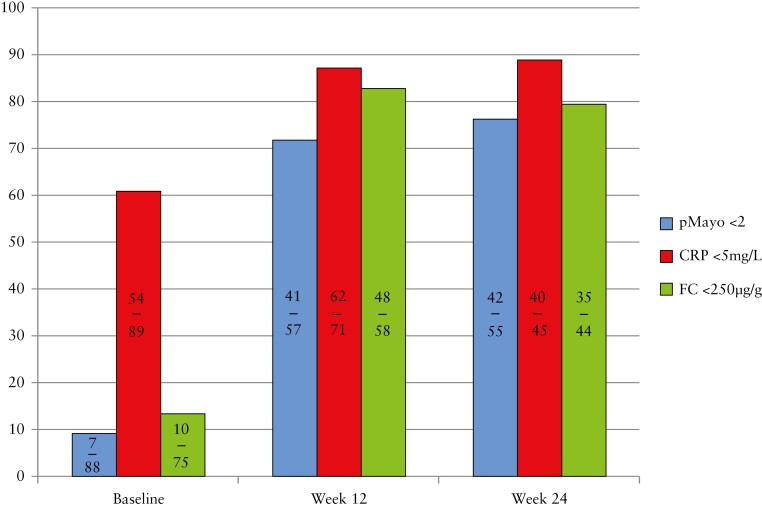
Clinical, biochemical, and faecal calprotectin biomarker remission at baseline, Week 12 and Week 24 from filgotinib initiation. CRP, C-reactive protein; FC, faecal calprotectin.

Clinical remission, biochemical remission, and faecal biomarker remission rates were 71.9% [41/57], 87.3% [62/71], and 82.8% [48/58] at Week 12, respectively [Figure 1]. A total of 65 patients [71.4%] had been on filgotinib for at least 24 weeks. Clinical remission, biochemical remission, and faecal biomarker remission were 76.4% [42/55], 88.9% [40/45], and 79.5% [35/44] at Week 24, respectively [Figure 1].

There was a significant reduction from baseline to Week 12 in median partial Mayo (5 [3-7] to 1 [0-2], *p *<0.0001), CRP (3 [1-11] to 1 [1-3], *p* <0.0001), and FC (780 [425-1166] to 107 [25-216], p <0.0001) [Table 1], whereas there was no significant change in the levels of total cholesterol and alanine transaminase [*p *= 0.25 and 0.15, respectively].

An exploratory analysis was done to identify factors associated with clinical remission at Week 12, but no factors were identified [variables analysed, Supplementary Table 1].

### 3.3. Filgotinib persistence

**Figure 2:**
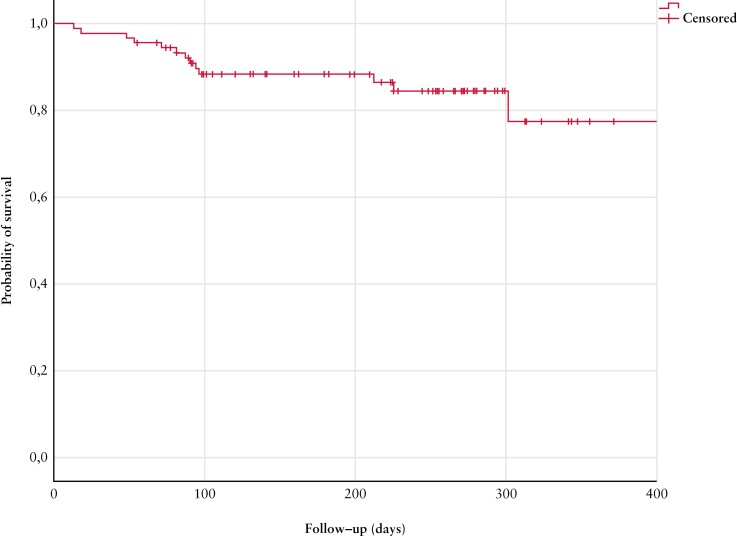
Kaplan–Meier curve for filgotinib persistence.

Median follow-up was 42 weeks [27-50]. At the end of follow-up 82.4% [75/91] patients remained on filgotinib, and median time to filgotinib discontinuation was 13 weeks [9-32] [Figure 2].

Reasons for drug discontinuation included: primary non-response 9.9% [9/16], pregnancy: 1.1% [1], adverse events: 1.1% [1], death: 1.1% [1], patient’s decision due to mental health reasons: 1.1% [1], ankylosing spondylitis not responding to filgotinib 1.1% [1], and secondary loss of response 2.2% [2]. Univariable and multivariable analysis were performed identifying decreased albumin levels [<36 g/L] at baseline associated with worse drug persistence (hazard ratio 4.71 [95% confidence interval 1.44-15.47], *p *= 0.011].

### 3.4. Steroid prescription and hospitalisation

During follow-up, 7 [7.7%] patients required steroid prescription after the induction period. Median time from filgotinib initiation to steroid course for these patients was 26 weeks [13-28]. Of those who required steroids during follow-up, all of them remained on filgotinib at the end of follow-up. At last observation only 3.8% [3/78] of the patients who remained on filgotinib were on steroids.

Finally, three [3.3%] patients needed hospital admission due to acute-severe ulcerative colitis [*n* = 1] or flare [*n *= 2]. Of note, one of these flares occurred after the patient was off therapy for 3 weeks due to issues with home care delivery. There were no colectomies during this initial period of follow-up. Median time from filgotinib initiation to hospitalisation was 6 weeks [3-13]. Among those needing hospital admission, only the one with acute-severe UC withdrew the drug.

### 3.5. Safety

Adverse events were documented in 15 patients [16.5%]. Mild adverse events occurred in 5.5% [5/91] including: headache, fatigue, mild nausea, and joint pain. Moderate adverse events leading to temporary discontinuation of filgotinib occurred in 8.8% [8/91] including: five respiratory tract infections, one facial shingles, and two mild COVID infections. Serious adverse events leading to definitive drug discontinuation or death occurred in two patients. One had a Stevens–Johnson syndrome that occurred 2 weeks after filgotinib initiation while the patient was taking also metronidazole for a dental infection. The other patient died from a biliary sepsis in the context of a disseminated malignancy that was unknown at time of commencing filgotinib.

## 4. Discussion

We present the first, real-world cohort investigating the effectiveness and safety of filgotinib in patients with UC, providing valuable contributions to the existing data from clinical trials. Our cohort is particularly noteworthy as it comprises a substantial proportion of biologic/small molecule-naïve patients, known to exhibit better responses to therapies compared with those previously exposed to such treatments. The data we have gathered demonstrate filgotinib’s efficacy in a relatively treatment-naïve UC patient population with predominantly moderately active disease, with significant improvements in clinical, biochemical, and faecal biomarker parameters observed at Week 12, which were further maintained through Week 24. Notably, clinical, biochemical, and faecal calprotectin remission at Week 12 was achieved in 71.9%, 87.3%, and 82.8% of patients, respectively, with a persistence rate exceeding 85% at 6 months. These findings are particularly encouraging, as they translate into meaningful outcomes for UC patients, with a substantial number of individuals experiencing the normalizsation of symptoms, inflammatory biomarkers, or both.

It is important to acknowledge that randomised trials represent the optimal approach for studying therapy efficacy and safety. However, such trials are often conducted in a selective and controlled manner to ensure high internal validity, which may raise concerns about the generalisability of the findings to ethnically diverse and heterogeneous populations.^11^ Our real-world cohort study serves as a valuable complement to randomised trials, addressing this limitation and providing insights into filgotinib’s effectiveness in a broader clinical setting.

The SELECTION trial, which evaluated filgotinib’s efficacy in UC, employed an ambitious primary endpoint including endoscopy assessment, setting a high standard for evaluating the drug’s performance as compared with previous small-molecule trials.^12–14^ The primary endpoint was defined as clinical remission considered as Mayo endoscopic subscore of 0 or 1, with rectal bleeding subscore of 0 and at least 1 point decrease in stool frequency from baseline for a subscore of 0 or 1 at Week 10.^2^ At Week 10, they found that 26.1% of biologic-naïve and 11.5% of biologic-experienced patients on filgotinib 200 mg met the endpoint. Whereas the endoscopy assessment at Week 12 limits the direct comparison of results between our study and the SELECTION trial, it reflects the pragmatic nature of real-world data, where such evaluations might not be feasible or practical in every patient.

Nonetheless, a post hoc analysis from the SELECTION trial found rapid and sustained symptom relief with filgotinib. Partial Mayo score was used although remission was considered with a partial Mayo ≤2 as compared with our study, where we defined clinical remission as partial Mayo <2. In our study, 71.9% of our patients achieved clinical remission by Week 12 as compared with 53.9% [bionaïve in SELECTION] and 33.2% [bio-experienced in SELECTION] at Week 10.^3^

Identifying predictive factors for therapy persistence in IBD is of paramount importance. Our study yielded low albumin levels [<36 g/L] as associated with lower drug persistence. Although the small number of drug discontinuation could be an explanation for the lack of further associations, low albumin levels are often seen in more severe cases of UC. In fact, hypo-albuminemia has served as a prognostic marker in other studies and it has been associated with increased clearance of infliximab.^15^ Further investigations with larger cohorts are essential to delve deeper into predictive factors and enhance our understanding of filgotinib’s long-term efficacy and response.

One of the favourable characteristics of filgotinib as compared with tofacitinib is its specific affinity to JAK1, which was hypothesised to induce fewer side effects. In our study, 15 patients [16.5%] had any adverse event and most of them were of mild or moderate severity, as compared with the SELECTION trial where approximately 60% of the patients had any adverse events with no differences between the drug and placebo.^2^ These differences could respond to the length of follow-up and to the retrospective nature of our study that may have influenced the reporting of mild side effects. Notably, no differences in total cholesterol levels were observed in our cohort, whereas a small increase in lipids levels was reported in the SELECTION trial after induction. One possible explanation for this is that 15.4% [14/91] of our cohort had cholesterol levels ≥5.9 mmol//L prior starting filgotinib, and were started on statins at that stage with consequent decrease of cholesterol levels by Week 12.

Filgotinib use in the UK has garnered acceptance among clinicians owing to its competitive pricing, which is closer to that of anti-TNF biosimilars than other new agents. This positive reception is further facilitated by its convenience as an oral medication that can be prescribed during clinic appointments and initiated on the same day, with fewer side effects than repetitive courses of steroids and faster onset of action as compared with thiopurines, with no need to attend infusion units reducing health care resources. Our cohort showcases data wherein the majority of patients exhibit moderately active disease in the context of a steroid-dependent or steroid-refractory setting. Previously, commencement of a thiopurine would be considered in this scenario. However, it is plausible that JAK inhibitors may change this drug positioning from this point onwards, given the efficacy and decreased need to monitor and titrate filgotinib as compared with thiopurines.

Our study has several strengths, including a diverse patient population and contributions to the current knowledge of JAK1 inhibition in UC. Nonetheless, there are some limitations to our study. First, this is a retrospective study and this could have impacted on the number of side effects recorded and data collection. There were also incomplete data for effectiveness outcomes. Finally, the follow-up was short although over 50% of the cohort was exposed to the drug for at least for 6 months. This time period still remains too early to draw firm conclusions about the medium to long-term efficacy and safety of maintenance therapy with filgotinib in UC.

Our study highlights the importance of real-world data in complementing findings from randomised trials. Future studies are needed, focusing on longer follow-up and trying to find potential predictive factors of better response that would strengthen the evidence supporting filgotinib’s use in UC therapy.

In conclusion, this real-world cohort study provides valuable insights into the effectiveness and safety of filgotinib in patients with UC in a cohort with a majority of biologic/small molecule-naïve patients and moderately active disease. The findings demonstrate that filgotinib effectively induces and maintains remission in UC patients, with favourable clinical, biochemical, and faecal biomarker responses persisting up to 6 months, with a favourable safety profile observed.

## Supplementary Material

jjad187_suppl_Supplementary_Material

## Data Availability

All data are incorporated into the article and its online Supplementary material.
