# Supplementary material for: Real-world Cohort Study on the Effectiveness and Safety of Filgotinib Use in Ulcerative Colitis
Source: J Crohns Colitis. 2023 Dec 8;19(1):jjad187. doi: 10.1093/ecco-jcc/jjad187 (PMC11760993; doi:10.1093/ecco-jcc/jjad187)
Supplement: jjad187_suppl_Supplementary_Material [file jjad187_suppl_supplementary_material.docx]

**Supplementary material**

**TABLE 1.**

| **Variables evaluated in the univariable analysis** |
| --- |
| Gender |
| Age at diagnosis |
| Disease extension |
| Disease duration |
| Previous thiopurine exposure |
| Previous TNF alfa inhibitor exposure |
| Previous vedolizumab exposure |
| Previous ustekinumab exposure |
| Previous tofacitinib exposure |
| Extra intestinal manifestations |
| C-Reactive Protein at baseline |
| Faecal calprotectin at baseline |
| Albumin level at baseline |
| Partial Mayo score at baseline |
| Concomitant steroids at baseline |
| Concomitant 5ASA at baseline |

**TABLE 2.**

**Univariable and multivariable analysis of predictive factors associated to drug persistence**

| **Variable** | **Univariable Cox Regression** | | | **Multivariable Cox Regression** | | |
| --- | --- | --- | --- | --- | --- | --- |
|  | Hazard Ratio | 95% CI | *p* | Hazard Ratio | 95% CI | *p* |
| Previous ustekinumab | 4.40 | 0.98-19.71 | 0.053 |  |  |  |
| Albumin <36gr/L | 5.23 | 1.64-16.72 | 0.005 | 4.71 | 1.44-15.47 | 0.011 |

Included in the multivariable analysis the variables whose p<0.1 in the univariable analysis.
